# MT-7716, a novel selective nonpeptidergic NOP receptor agonist, effectively blocks ethanol-induced increase in GABAergic transmission in the rat central amygdala

**DOI:** 10.3389/fnint.2014.00018

**Published:** 2014-02-18

**Authors:** Marsida Kallupi, Christopher S. Oleata, George Luu, Koji Teshima, Roberto Ciccocioppo, Marisa Roberto

**Affiliations:** ^1^Committee on the Neurobiology of Addictive Disorders, The Scripps Research Institute, La JollaCA, USA; ^2^Pharmacology Unit, School of Pharmacy, University of CamerinoCamerino, Italy; ^3^Department II (CNS), Pharmacology Research Laboratories I, Research Division, Mitsubishi Tanabe Pharma CorporationYokohama, Japan

**Keywords:** amygdala, GABA, alcohol, nociceptin, NOP receptor, electrophysiology

## Abstract

The GABAergic system in the central amygdala (CeA) plays a major role in ethanol dependence and the anxiogenic-like response to ethanol withdrawal. A large body of evidence shows that Nociceptin/Orphanin FQ (N/OFQ) regulates ethanol intake and anxiety-like behavior. In the rat, ethanol significantly augments CeA GABA release, whereas N/OFQ diminishes it. Using electrophysiological techniques in an *in vitro* slice preparation, in this study we investigated the effects of a nonpeptidergic NOP receptor agonist, MT-7716 [(*R*)-2-3-[1-(Acenaphthen-1-yl)piperidin-4-yl]-2-oxo-2,3-dihydro-1*H*-benzimidazol-1-yl-*N*-methylacetamide hydrochloride hydrate], and its interaction with ethanol on GABAergic transmission in CeA slices of naïve rats. We found that MT-7716 dose-dependently (100–1000 nM) diminished evoked GABA_A_ receptor-mediated inhibitory postsynaptic potentials (IPSPs) and increased paired-pulse facilitation (PPF) ratio of these evoked IPSPs, suggesting a presynaptic site of action of the MT-7716 by decreasing GABA release at CeA synapses. The presynaptic action of MT-7716 was also supported by the significant decrease in the frequency of miniature inhibitory postsynaptic currents (mIPSCs) induced by the nociceptin receptor (NOP) agonist.** Interestingly, MT-7716 prevented the ethanol-induced augmentation of evoked IPSPs. A putative selective NOP antagonist, [Nphe1]Nociceptin(1–13)NH2, totally prevented the MT-7716-induced inhibition of IPSP amplitudes indicating that MT-7716 exerts its effect through NOPs. These data provide support for an interaction between the nociceptin and GABAergic systems in the CeA and for the anti-alcohol properties of the NOP activation. The development of a synthetic nonpeptidergic NOP receptor agonist such as MT-7716 may represent a useful therapeutic target for alcoholism.

## Introduction

Alcoholism is a chronically relapsing disorder characterized by compulsive drug- seeking and taking (Koob and Le Moal, 1997). It is one of the most prevalent health problems worldwide; nevertheless there are very few medications available for treating it. Understanding the neurobiology of alcohol abuse and addiction will strongly contribute to the development of effective new pharmacotherapies for alcoholism. Recently, a body of research has been focused on the identification of new targets for pharmacological treatments of alcohol addiction; among these, several peptidergic systems known for their established role in the regulation of stress response and anxiety-like behaviors associated with the development of alcohol addiction.

Nociceptin/Orphanin FQ (N/OFQ) is an opioid-like peptide (Meunier et al., 1995; Reinscheid et al., 1995; Meunier, 1997), that acts at opioid-like receptors (Calo et al., 2000), although it does not bind to classic opioid receptors. N/OFQ and other NOP agonists have shown an anxiolytic-like profile in animal studies (Jenck et al., 1997, 2000). It decreases alcohol drinking, and prevents relapse-like behavior in rats (Ciccocioppo et al., 2000, 2002b, 2004, 2007; Kuzmin et al., 2007; Ubaldi et al., 2013).

Central intracranial injection of N/OFQ is demonstrated to induce anxiolytic-like effects in several behavioral paradigms, each generating different types of anxiety leading to the theory that this peptide may act as an endogenous regulator of acute anxiety. Studies in knockout animals have shown that genetically engineered nociceptin precursor-deficient mice display an increased susceptibility to acute and repeated stress, as compared to their wild-type littermates (Koster et al., 1999; Reinscheid et al., 1999). Moreover, N/OFQ inhibits stress-induced ethanol seeking and attenuates various extrahypothalamic effects of corticotropin releasing factor (CRF), the major mediator of stress in mammals (Allison and Sheehy, 1992; Ciccocioppo et al., 2002a, 2004; Martin-Fardon et al., 2010; Schank et al., 2012). In Wistar rats with a history of ethanol dependence, neuroadaptive changes in the N/OFQ system have been associated with increased stress sensitivity and alcohol intake (Braconi et al., 2010; Aujla et al., 2013), as well as a more pronounced anxiolytic effect of N/OFQ in dependent rats in comparison to naïve rats. It has been well documented that systemic administration of alcohol alters basal levels of N/OFQ in several brain regions, as well as mRNA expression in animals previously exposed to stress (Roberto and Siggins, 2006; Higley et al., 2012). In addition to these evidences, our laboratory has previously reported at the cellular level that N/OFQ dose-dependently decreases evoked and spontaneous GABA_A_-mediated transmission in the central amygdala (CeA) decreasing presynaptic GABA release (Roberto and Siggins, 2006). Importantly, in CeA from ethanol-dependent rats the N/OFQ-induced decrease in CeA GABAergic transmission is larger than that observed in naïve rats, suggesting that neuroadaptations occur at these synapses during chronic alcohol exposure (Roberto and Siggins, 2006). Notably, the CeA has been also identified as the putative brain site of action of N/OFQ for its inhibitory effects on ethanol drinking (Economidou et al., 2008).

Jenck et al. (2000) developed the first nonpeptidergic brain-penetrant NOP receptor agonist, Ro 61-6198, that was tested on alcohol-related behaviors (Kuzmin et al., 2007) and (Economidou et al., 2006). Another small-molecule NOP agonist, 2-3-[1-((1R)-acenaphthen-1-yl)piperidin-4-yl]-2,3-dihydro-2-oxo-benzimidazol-1-yl-N-methylacetamide (W-212393), was synthesized by Teshima et al. (2005) and tested in rats on the circadian body temperature rhythm of rats. Recently, a blood brain barrier penetrating NOP receptor agonist MT-7716, hydrochloride of W-212393 has become available, providing a suitable pharmacological tool to study the treatment target potential of the nociceptin system with direct translational implications. MT-7716 has high affinity for human NOP receptors expressed in HEK293 cells. The affinity of MT-7716 for the NOP is almost equal to that of the endogenous agonist N/OFQ, and higher than that of other nonpeptidergic NOP agonist, Ro 64-6198. NOP agonistic activities of MT-7716 were evaluated by GTPγ35S binding to human NOP expressed in HEK293 cells and the maximum effect was almost equal to that of N/OFQ, suggesting that MT-7716 is a full agonist for NOP receptors (Teshima personal communication).

Here, we investigated the effect of this novel molecule *per se* on the CeA GABAergic transmission and its interaction with acute ethanol application in CeA slices from naïve control rats. Similar to our previous electrophysiological studies (Roberto and Siggins, 2006; Cruz et al., 2012) on the characterization of N/OFQ actions in rat CeA, we found that MT-7716 dose-dependently decreases GABAergic transmission and effectively blocks the ethanol-induced increase in GABA release at these synapses. Our studies provide insights in the underlying mechanisms of MT-7716 effects on the GABAergic transmission in the CeA and support the importance of developing nonpeptidergic NOP agonists, as valid pharmacological tools to treat alcoholism.

## Materials and methods

### Animals

Male Wistar rats (*n* = 70) (Charles River, Wilmington, MA, USA), at the age of 8–12 weeks were used. Their body weight ranged between 330 and 370 g at the time of slice-recordings. Rats were housed two per cage in a room with reversed artificial 12:12 h light/dark cycle (lights off at 8:00 A.M.) at constant temperature (20–22°C) and humidity (45–55°), with *ad libitum* access to tap water and food pellets (PJ Noyes Company, Inc., Lancaster, NH). All procedures met the guidelines of *The Scripps Research Institute IACUC and NIH guidelines on the care and use of laboratory animals*.

### Electrophysiological studies

#### Slice preparation

CeA slices were prepared as previously described, (Roberto et al., 2003, 2004b; Cruz et al., 2012), from rats anesthetized with isoflurane (1–3%) and immediately decapitated. Transverse slices were cut 300–400 μm thick on a Leica 1000S vibratome (Campden, Lafayette, Indiana). They were incubated in an interface configuration for about 20 min, completely submerged and continuously superfused in cold gassed artificial cerebrospinal fluid (ACSF) of the following composition (in mM): NaCl, 130; KCl, 3.5; NaH_2_PO_4_, 1.25; MgSO_4_•7H2O, 1.5; CaCl_2_, 2.0; NaHCO_3_, 24; glucose, 10. Drugs were added to the warm (31°C) ACSF, (flow rate of 2–4 ml/min) from stock solutions to obtain known concentrations in the superfusate.

#### Intracellular recording of evoked responses

Recordings were performed from CeA neurons (from the medial subdivision of the CeA) with sharp micropipettes filled with 3M KCl using discontinuous current-clamp mode, (Roberto et al., 2004b; Haubensak et al., 2010; Cruz et al., 2012). Most neurons were held near their resting membrane potential (RMP). Data were acquired with an Axoclamp-2A preamplifier (Axon Instruments, Foster City, CA) and stored for later analysis using pClamp software (Axon Instruments, Foster City, CA). We evoked pharmacologically-isolated GABA_A_ receptor-mediated inhibitory postsynaptic potentials (IPSPs) by stimulating locally within the CeA through a bipolar stimulating electrode while superfusing the slices with the glutamate receptor blockers 6,7-Dinitroquinoxaline-2, 3-dione (DNQX; 20 μM) and DL-2-amino-5-phosphonovalerate (APV; 30 μM), and the GABA_B_ receptor antagonist ((3-N[1-(S)-(3,4-Dichlorophenyl)ethyl]amino-2-(S)-hydroxypropyl)-benzyl-phosphinic acid (CGP) 55845A; 1 μM). At the end of the recording, superfusion with either 30 μM bicuculline or 50 μM picrotoxin was routinely performed to confirm the GABA_A_ergic nature of the IPSPs. To determine the synaptic response parameters for each cell, we performed an input-output (I-O) protocol (Roberto et al., 2003, 2004b) consisting of a range of five current stimulations (50–250 mA; 0.125 Hz), starting at the threshold current required to elicit an IPSP up to the strength required to elicit the maximum amplitude. These stimulus strengths were maintained throughout the entire duration of the experiment. In our graphs only the three middle intensities are plotted. The synaptic responses were quantified by calculating the IPSP amplitude with Clampfit software (Axon Instruments). The paired-pulse facilitation (PPF) in each neuron was examined by using paired stimuli at 50 and 100 ms inter-stimulus interval (Roberto et al., 2004b). The stimulus strength was adjusted such that the amplitude of the first IPSP was 50% of maximal, determined from the I-O relationship. The PPF ratio was calculated as the second IPSP amplitude over that of the first IPSP.

#### Whole-cell patch-clamp recording of miniature inhibitory postsynaptic currents (mIPSCs)

We recorded from CeA neurons visualized in brain slices (300 μm) using infrared differential interference contrast (IR-DIC) optics and CCD camera (EXi Aqua, QImaging) (Gilpin et al., 2011; Cruz et al., 2012; Herman et al., 2013). A w60 water immersion objective (Olympus) was used to identify and approach the CeA neurons. Whole-cell voltage-clamp recordings were made with a Multiclamp 700B amplifier (Molecular Devices), low-pass filtered at 2–5 kHz, digitized (Digidata 1440A; Molecular Devices), and stored on a PC using pClamp 10 software (Axon Instruments). All voltage-clamp were performed in a gap-free acquisition mode with a sampling rate per signal of 10 kHz. Patch pipettes (4–8 M’Ω) were pulled from borosilicate glass (Warner Instruments) and filled with an internal solution composed of (in mM): 145 KCl; 0.5 EGTA; 2 MgCl_2_; 10 HEPES; 2 Na-ATP; 0.2 Na-GTP. GABAergic miniature IPSCs (mIPSCs) were recorded in the presence of 20 μM DNQX, 30 μM DL-AP5, 1 μM CGP 55845A and 1 μM tetrodotoxin (TTX). Drugs were constituted in ACSF and applied by bath superfusion. All 12 cells were clamped at −60 mV for the duration of the recording. In all experiments, series resistance (<10 M’Ω) was continuously monitored with a 10 mV hyperpolarizing pulse and experiments with >20% changes in series resistance were not included in final analysis. Frequency, amplitude and kinetics of mIPSCs were analyzed using a semi-automated threshold based mini detection software (Mini Analysis, Synaptosoft Inc., Fort Lee, NJ) and were visually confirmed. To accurately determine the mIPSC amplitude, only mIPSCs with >5 pA were accepted for analysis. The choice of this cutoff amplitude for acceptance of mIPSCs was made to obtain a high signal-to-noise ratio. Averages of mIPSC characteristics were based on a minimum time interval of 3–5 min and a minimum of 50 events. All detected events were used for event frequency analysis, but superimposed events were eliminated for amplitude and decay kinetic analysis. All data are expressed as mean ± SEM.

#### Drugs

CGP 55845A, DL-AP5, picrotoxin and bicuculline were purchased from Sigma (St. Louis, MO), TTX was purchased from Biotum (Hayward, CA); DNQX and [Nphe1]Nociceptin(1–13)NH2 from Tocris (Ellisville, MO) and ethanol from Remet (La Mirada, CA). MT-7716, (*R*)-2-3-[1-(Acenaphthen-1-yl)piperidin-4-yl]-2-oxo-2,3-dihydro-1*H*-benzimidazol-1-yl-*N*- methylacetamide hydrochloride hydrate, was synthesized at Mitsubishi Tanabe Pharma Corporation (Japan). It was dissolved in distilled water.

#### Data analysis and statistics

To analyze data acquired from intracellular and whole cell recordings, Clampfit 8.2 (Molecular Devices) and MiniAnalysis 5.1 software (Synaptosoft, Leonia, NJ) were respectively used.

GraphPad Prism 5.0 software (GraphPad Software, San Diego, CA) and Statistica Package were employed for all statistical analysis of results obtained by intracellular recording and for figure presentations. Statistical significance was set at *p* < 0.05 level, using one-way ANOVA, Student’s *t*-test or one-sample *t*-test/Wilcoxon signed rank test. *T*-test analysis was used for individual means comparisons and within-subject one-way repeated measures (RM) ANOVA to compare IPSPs within a group. When appropriate, Newman-Keuls *post-hoc* test was used to assess significance between treatments with *p* ≤ 0.05 considered significant.

The mIPSC results were evaluated with cumulative probability analysis, and statistical significance was determined using the Kolmogorov-Smirnov, non-parametric two-sample test with *p* < 0.05 considered significant for each neuron. The pooled data from all 12 CeA neurons studied were then analyzed by paired *t*-test analysis for individual means comparisons to evaluate MT-7716 effects.

## Results

### MT-7716 decreased evoked GABAergic transmission in central amygdala(CeA) neurons

**Figure 1:**
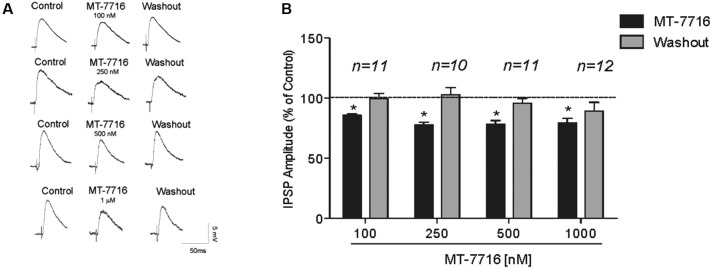
**MT-7716 decreases evoked GABAergic transmission in CeA neurons. (A)** *Left panel*: Representative recordings of evoked IPSPs in CeA neurons from naïve rats recorded before, during, and after washout from application of MT-7716 at all the concentrations tested. **(B)** *Right Panel*: Histograms representing the percent of the peak decrease in evoked (at half max stimulus intensity) IPSP amplitudes during superfusion of different concentrations (100, 250, 500, and 1000 nM) of MT-7716 and washout. Overall ANOVA revealed that MT-7716 decreased statistically significantly the IPSP amplitudes. Post hoc Newman-Keuls showed significant effect for all the doses at half max stimulus intensity. (*) Indicates *p* < 0.01.

**Figure 2:**
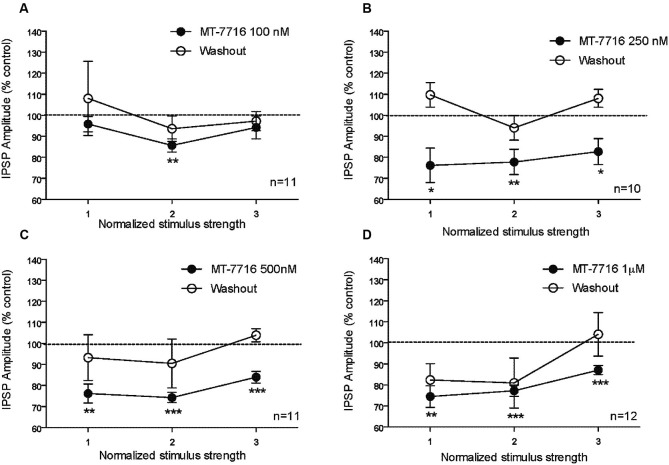
**The percentage effect of MT-7716 on the IPSP amplitude for the three middle stimulus intensities. (A)** In the CeA of control rats, MT-7716 100 nM significantly (** *p* < 0.01) decreases the mean amplitude of evoked IPSP over the middle stimulus strength intensity tested (*n* = 11). **(B)** MT-7716 250 nM significantly decreases the mean amplitude of evoked IPSP over the three middle stimulus strength intensities tested (*n* = 10) (* *p* < 0.05) and (** *p* < 0.01). **(C–D)** MT-7716 500 and 1000 nM significantly decrease the mean amplitude of evoked IPSPs over the three middle stimulus strength intensities tested (*n* = 11/12) (** *p* < 0.01) and (*** *p* < 0.001). All data are expressed as % of control for three normalized stimulus strengths. Student *t*-test was used to analyze the percentage effect of MT-7716 on the IPSP amplitude.

We recorded from 81 CeA neurons from male Wistar rats. The mean RMP was −78 ± 1.7 mV and the mean input resistance was 115 ± 5 M. We evoked pharmacologically isolated GABA_A_-IPSPs by stimulating locally within the CeA and IPSP input-output (I/O) curves were generated. Based on our previous electrophysiological data on N/OFQ (Roberto and Siggins, 2006) we generated a dose-response curve testing four ranged concentrations (100 nM, 250 nM 500 nM and 1 μM) of MT-7716 on the mean amplitude of evoked IPSPs in CeA neurons from naïve-control rats (Figures 1A, B). We applied MT-7716 on CeA slices for 15–20 min and washed out for more than 25 min, until partial or complete recovery was obtained. In Figure 1B, we expressed the data as percent of control using the middle stimulus intensity obtained from the I-O relationship. The graphs in Figures 2A–D plot the percentage effect of MT-7716 on the IPSP amplitude for the three stimulus intensities and the washout.

Although, the lowest (100 nM) concentration of MT-7716 tested, only slightly decreased the mean amplitude of evoked IPSPs to 91 ± 4% of control (*n* = 11, Figure 2A) over the three middle intensities, it did significantly decrease the amplitude of IPSPs evoked by the half maximal intensity. Notably, 250 nM MT-7716 significantly decreased the amplitude of evoked IPSPs to 78 ± 7% (*n* = 10) with complete recovery after washout (Figure 2B). Similarly, in another 11 CeA neurons, application of 500 nM MT-7716 decreased significantly the mean evoked IPSP amplitudes to 78 ± 3% (Figure 2C). This MT-7716 induced decrease of evoked IPSP amplitude was reversible after washout in all the above listed experiments. The highest concentration of MT-7716 tested (1 μM), significantly decreased the mean amplitude of evoked GABA IPSPs to 80 ± 3% of control over the three-stimulus intensities in 12 cells (Figure 2D).

**Figure 3:**
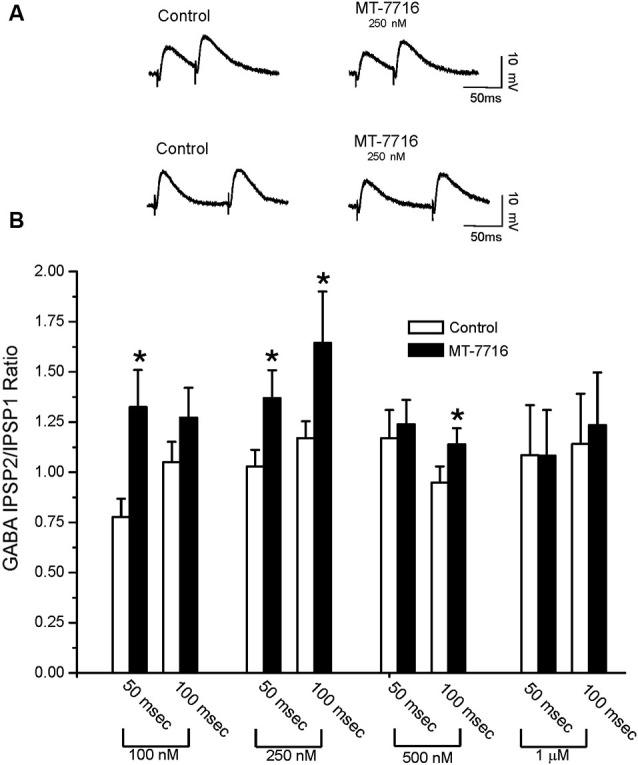
**MT-7716 decreases GABAergic transmission in CeA neurons by decreasing GABA release. (A)** Representative recordings of PPF at both 50 (upper traces) and 100 (lower traces) ms in a CeA neuron from naïve rat before and during superfusion of 250 nM MT-7716. **(B)** Overall ANOVA revealed that MT-7716 (100 and 250 nM) significantly increases the PPF ratio of evoked IPSPs using 50 ms interstimulus intervals. MT-7716 (250 and 500 nM) significantly increases the PPF ratio of evoked IPSPs using 100 ms interstimulus intervals. (*) Indicates (*p* < 0.05) after appropriate *Post-hoc* Newman-Keuls test.

To evaluate whether the effect of MT-7716 was occurring at the pre- or postsynaptic locus, we determined changes in PPF ratio, a measure inversely related to neurotransmitter release (Andreasen and Hablitz, 1994; Bonci and Williams, 1997; Roberto et al., 2003). In brief, in CeA neurons, 100 nM MT-7716 significantly (*n* = 8; *p* < 0.05) increased 50 ms PPF ratio from 0.77 ± 0.09 to 1.31 ± 0.18 and slightly increased the 100 ms PPF ratio from 1.04 ± 0.10 to 1.26 ± 0.14 (Figures 3A, B). The intermediate dose 250 nM MT-7716 significantly increased both 50 and 100 ms PPF ratio from 1.02 ± 0.08 and 1.2 ± 0.08 to 1.36 ± 0.13 and 1.63 ± 0.25 respectively, (*p* < 0.05 and *p* < 0.04), suggesting decreased GABA release. MT-7716 500 nM did not alter the 50 ms PPF ratio (baseline 1.16 ± 0.14; MT-7716 1.23 ± 0.12; *n* = 8), but increased significantly the 100 ms PPF ratio (*p* < 0.05) from 0.94 ± 0.08 to 1.13 ± 0.08; *n* = 6). In 7 CeA neurons, MT-7716 (1000 nM) did not alter either PPF ratio 50 or PPF ratio 100 ms. (PPF 50 ms: baseline 1.07 ± 0.24; MT-7716 1.07 ± 0.22; PPF 100 ms: baseline 1.13 ± 0.24; MT-7716 1.22 ± 0.26). In summary, we found that MT-7716 at the doses of 100, 250 and 500 nM significantly increased PPF ratios.

**Figure 4:**
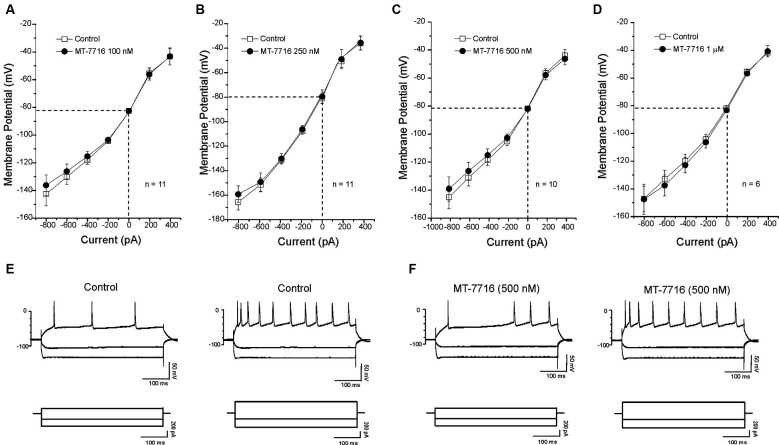
**MT-7716 has no effect on voltage-current relationships on the CeA neurons. (A–D)** I/V curves showing that MT-7716, in all doses superfused (100–1000 nM) Overall ANOVA indicates that MT-7716 does not modify the RMP of the CeA neurons (*n* = 6–11). **(A)** The mean RMPs for the neurons tested with 100 nM MT-7716 was −81 ± 1.2 mV and was −80 ± 0.5 mV for those tested 250 nM MT-7716 **(B)**. Similarly the RMPs of the 10 and 6 CeA neurons tested with 500 nM and 1000 nM MT-7716 was −81.5 ± 0.9 mV **(C)** and −81 ± 1.2 mV **(D)**. **(E)** Representative current clamp recordings of a CeA neuron (RMP = 80 mV; input resistance 113 M) during control and 500 nM MT-7716 superfusion **(F)**. Overall, MT-7716 did not significantly affect the firing pattern or number of action potentials in our CeA neuronal population.

We also evaluated if different concentrations of MT-7716 would affect the passive membrane properties of CeA neurons of male Wistar rats. Similar to our N/OFQ studies in Sprague Dawley rats (Roberto and Siggins, 2006), we found that none of the concentrations of MT-7716 used, altered the resting membrane properties (Figures 4A–D). Current–voltage (I–V) relationship analysis showed that MT-7716 at the four concentrations tested had no significant effect on (RMP), conductance (Figures 4A–D), or the number of action potentials upon depolarization across the CeA neurons (Figures 4E, F). The mean of the RMPs and input resistance of the four groups of CeA neurons tested in the dose-dependent study was 80.7 ± 1.5 mV and 117 ± 7.6 MΩ, respectively. Specifically, the number of actions potentials for neurons in response to 200 and 400 pA current injections were: 3.2 ± 1.4 and 9.7 ± 1.8 during control and 3.1 ± 1.5 and 9.2 ± 1.8 during 100 nM MT-7716; 4.6 ± 1.1 and 11.8 ± 1.1 during control and 4.5 ± 1.1 and 12.2 ± 1.4 during 250 nM MT-7716; 4.1 ± 0.9 and 10.9 ± 1.7 during control and 4.3 ± 1.6 and 11.3 ± 2.1 during 500 nM MT-7716; 2.5 ± 1.5 and 8.3 ± 2.4 during control and 2.5 ± 1.6 and 8.3 ± 2.8 during 1000 nM MT-7716. Representative current clamp recordings from a CeA neuron during control conditions (Figure 4E) and application of 500 nM MT-7716 (Figure 4F) are illustrated in Figure 4.

### MT-7716 decreased spontaneous miniature inhibitory postsynaptic currents (mIPSCs) in central amygdala (CeA)

**Figure 5:**
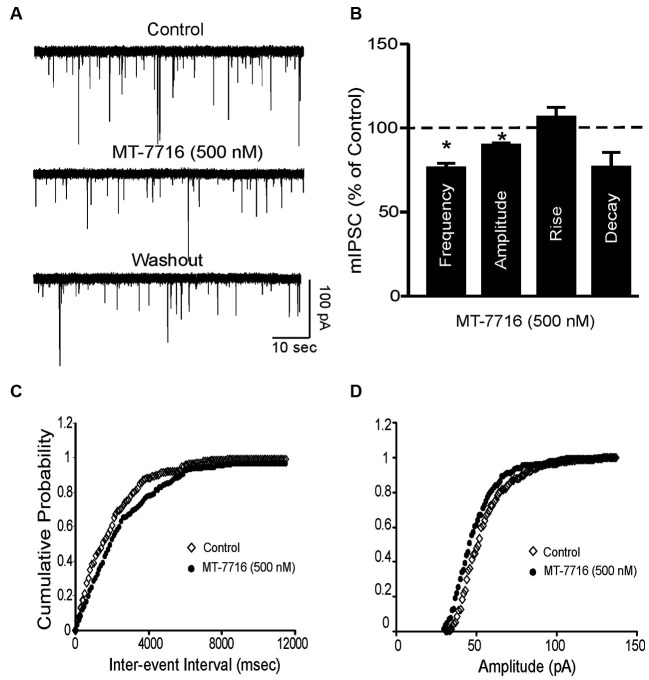
**MT-7716 decreased spontaneous miniature inhibitory postsynaptic currents (mIPSCs) in CeA. (A)** Representative CeA mIPSCs before, during the superfusion of 500 nM MT-7716 and washout. **(B)** Mean ± SEM frequency, amplitude, rise and decay of mIPSCs for CeA neurons from control rats. MT-7716 significantly (* *p* < 0.001) decreased the mean mIPSC frequencies and amplitude. Statistical significance * was set at *p* < 0.05 and calculated by Student’s *t*-test. **(C)** Cumulative fractions calculated by Kolmogorov-Smirnov sample test show that MT-7716 shifted the cumulative frequency to the right (in 11 out of 12 CeA neurons studied), indicating a longer inter-event interval during its application, suggesting decreased GABA release. **(D)** Cumulative fractions calculated by Kolmogorov-Smirnov sample test show that MT-7716 shifted the cumulative frequency to the right (in 10 out of 12 CeA neurons studied). MT-7716 shifted the cumulative amplitude to the left, indicating smaller mIPSC amplitudes, suggesting postsynaptic site of action.

To further characterize the decreased GABA release induced by MT-7716, we examined spontaneous mIPSCs using whole-cell recordings in the presence of 1 μM TTX to eliminate action potential-dependent release of neurotransmitter. Here we tested MT-7716 at 500 nM, a maximal effective and reversible concentration, and found that MT-7716 significantly (*p* < 0.05) decreased mIPSC frequency to 78.9 ± 5.3% of control (means: control, 0.82 ± 0.3 Hz; MT-7716, 0.67 ± 0.3 Hz; *n* = 12) with recovery during washout (0.75 ± 0.4 Hz) (Figures 5A, D). MT-7716 significantly decreased the frequency of mIPSCs and shifted the cumulative frequency distribution to longer inter-event intervals (Figures 5A–C), indicating decreased presynaptic GABA release. MT-7716 also significantly (*p* < 0.05) decreased the amplitude to 90.15 ± 0.5% of control (means: control, 62.3 ± 2.7 pA; MT-7716, 56.06 ± 2.4 pA; *n* = 12; Figures 5A, B and D), but not the decay or rise time of mIPSCs.

### Interaction of MT-7716 and ethanol at the central amygdala (CeA) GABAergic synapses

**Figure 6:**
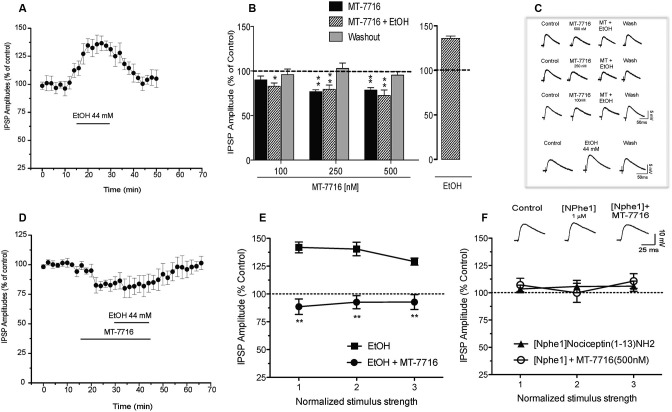
**Interactions of MT-7716 and ethanol at the CeA GABAergic synapses. (A)** Overall ANOVA for the analyze of the time course of the % IPSP amplitude in CeA neurons during ethanol application *per se* shows that ethanol significantly increases the amplitude of evoked IPSPs. **(B)** Histograms representing the percent of the peak decrease in evoked (at half max stimulus intensity) IPSP amplitudes during superfusion of MT-7716 at the concentrations (100, 250 and 500 nM) alone, and in the presence of ethanol 44 mM on top. Newman-Keuls *post-hoc* test showed that MT-7716 decreased significantly the evoked IPSP amplitudes and blocked the ethanol-induced facilitation. (*) Indicates (*p* < 0.05) (**) indicates (*p* < 0.01). **(C)** Representative evoked IPSPs recorded before and during MT-7716 (100–500 nM) and co application with ethanol and washout.** (D)** Time course of the application of MT-7716 (500 nM) that reduces the amplitude of evoked IPSPs. After 15–20 min of MT-7716 superfusion, co-application of ethanol does not increase the evoked IPSP amplitude (72.9 ± 1.1% of control). MT-7716 effectively blocks the ethanol-induced enhancement of IPSPs, and GABA transmission returned to baseline levels upon 25 min of washout (94 ± 10% of control). **(E)** Ethanol significantly (*p* < 0.05) increased (137.1 ± 4.7% of control) the evoked IPSPs and 500 nM MT-7716 in the presence of ethanol significantly (** *p* < 0.01 by Newman-Keuls *post-hoc* test) decreased (91.3 ± 1.4%) the IPSPs and blocked the ethanol-induced facilitation. **(F)** Application of [Nphe1]Nociceptin(1–13)NH2 alone did not alter evoked IPSPs (105.1 ± 4.6% of control); *n* = 7; by paired *t*-test but blocked the MT-7716-induced decrease of IPSPs.

We have previously reported (Roberto et al., 2003) that 44 mM ethanol (a maximally effective concentration) increases evoked GABA IPSPs via increased GABA release in the CeA. We have also documented that N/OFQ blocks this ethanol-induced facilitation (Roberto and Siggins, 2006). In the present study we recapitulate that ethanol 44 mM significantly (*p* < 0.05) and reversibly increases by 36 ± 3% (*n* = 6) the amplitude of evoked IPSPs (Figures 6A–C). This ethanol-induced increase is associated with a significant (*p* < 0.05) decrease in the PPF ratio of IPSPs (both 50 and 100 ms intervals; data not shown). We then examined whether MT-7716 would block the ethanol-induced increase in evoked GABAergic responses. In separate groups of CeA cells, we applied MT-7716 at the doses of 100, 250 and 500 nM and then co-applied 44 mM ethanol on the top (Figures 6B, C). In Figures 6B the data are expressed as percent of control using the three middle stimulus intensities (1–3X) obtained from the I-O relationship. All three concentrations of MT-7716 used (100, 250 and 500 nM) significantly decreased IPSP amplitudes (half maximal intensity) and totally blocked the ethanol-induced facilitation of IPSPs. Specifically, MT-7716 (500 nM) significantly (*p* < 0.001; *n* = 7) reduced the amplitude of evoked IPSPs by 20% of control over all stimulus strengths in the CeA neurons (Figure 6B). After 15–20 min of MT-7716 superfusion, co-application of ethanol (44 mM) did not increase the evoked IPSP amplitude (72.9 ± 1.1% of control). MT-7716 effectively prevented the ethanol-induced enhancement of IPSPs, and GABA transmission returned to baseline levels upon washout (94 ± 10% of control; Figure 6D). Of note is the fact that MT-7716 in lower doses, 250 and 100 nM also decreased evoked IPSPs to 79 ± 8% (*n* = 6) and 100 nM to 90 ± 6% (*n* = 6) of control respectively and blocked ethanol-induced increase of IPSPs (the IPSPs amplitude remained the same 80 ± 10% and 83 ± 3% of control, respectively) with total recovery on washout. Interestingly, although the lowest concentration of 100 nM MT-7716 had no significant effect on evoked IPSP amplitudes (*p* > 0.05) (10% decrease compared to control), it still completely blocked the ethanol-induced increase of IPSPs with total recovery on washout, suggesting that the anti-ethanol actions of NOP activation were not due simply to a summation of opposing effects, but functionally independent effects on GABA transmission. We did not test the highest concentration of MT-7716 because although the inhibition induced by 1000 nM MT-7716 was comparable to the one obtained with 500 and 250 nM, this effect was only partially recovered upon washout, data not shown.

To assess the effectiveness of MT-7716 in blocking the ethanol effects, we reversed the order of drugs application: we first applied ethanol and then added MT-7716. Acute application of ethanol significantly (*p* < 0.05) increased to 137.1 ± 4.7% of control the amplitude of evoked IPSPs over all stimulus strengths (Figure 6E) in 5 CeA neurons and decreased 50 and 100 ms PPF ratios from 1.21 ± 0.17 and 1.31 ± 0.14, to 0.85 ± 0.08 and 0.92 ± 0.02, respectively. Superfusion of MT-7716 500 nM in the presence of ethanol significantly reduced the mean evoked IPSP amplitude to 91.3 ± 1.4% of control with recovery upon washout. MT-7716 effectively blocked the ethanol-induced enhancement of IPSPs, and GABA transmission returned to baseline levels upon washout (103.3 ± 9.2% of control. MT-7716 application in the presence of ethanol, correlated with an increase in the 50 and 100 ms PPF ratio to 1.25 ± 0.13 and 1.37 ± 0.17, respectively. These effects returned to baseline values after washout.

To determine whether the MT-7716-induced inhibition of evoked CeA GABAergic transmission occurs via NOP activation we tested [Nphe1]Nociceptin(1–13)NH2, a putative selective NOP receptor antagonist (Roberto and Siggins, 2006; Cruz et al., 2012; Kallupi et al., 2013). Application of [Nphe1]Nociceptin(1–13)NH2 (1 μM) alone did not alter evoked IPSPs (105.1 ± 4.6% of control over the three middle intensities; *n* = 7; by paired *t*-test; Figure 6F). To verify that MT-7716 activate NOPs we applied 500 nM MT-7716 in the presence of the antagonist and found that MT-7716 no longer decreased the evoked IPSPs (Figure 6F). These data suggest lack of tonic activity of the endogenous NOPs and MT-7716 exerts its effect through NOPs. In 4 of the 7 cells, we also tested whether concomitant application of [Nphe1]Nociceptin(1–13)NH2 and MT-7716 affects ethanol-induced increases in evoked IPSPs. We found that MT-7716 in the presence of the NOP antagonist did not prevent the 44 mM ethanol-induced augmentation of IPSPs (135.2 ± 5.6% of control at half maximal stimulus intensity; *n* = 4; data not shown), confirming that NOP antagonism blocks the MT-7716 inhibition of ethanol-induced facilitation.

## Discussion

Alcohol consumption has been identified as an important risk factor for illness, disability, and mortality (Greenfield et al., 2009; Mohapatra et al., 2010). Because detoxification does not stop the craving for alcohol, in rats, like in humans, recovery is often difficult to maintain. There are a few drugs that have been FDA-approved to reduce alcohol craving like Acamprosate and Naltrexone (Koob et al., 2002; Mann et al., 2004; Dahchour et al., 2005; Mann et al., 2008; Umhau et al., 2011; Spanagel and Vengeliene, 2013), however the study of new therapeutics for alcoholism is still in progress. Several lines of evidence suggest that the N/OFQ system serves an important role in the regulation of various aspects of abused drugs and points to NOP receptor agonism as potentially beneficial for the treatment of anxiety and addictions (Lambert, 2008; Gavioli and Calo, 2013; Witkin et al., 2014).

The CeA, a nucleus predominantly composed of GABAergic inhibitory neurons, is essential for playing a role in negative reinforcement, in fact acute and chronic alcohol effects on brain stress systems can refer, among others, the recruitment of extrahypothalamic brain stress systems such as CeA (Koob, 2009; Martin-Fardon et al., 2010). We have previously documented that ethanol increases GABAergic synaptic transmission in the CeA via increased presynaptic GABA release (Roberto et al., 2003). Specifically, ethanol augments evoked inhibitory postsynaptic currents (IPSCs), decreases (PPF) of evoked IPSCs, and increases the frequency of miniature inhibitory postsynaptic currents (mIPSCs) in most CeA neurons, indicating that alcohol increases GABA release. These electrophysiological findings were also validated by *in vivo* microdialysis studies showing that *in vivo* administration of ethanol via microdialysis probe produced a dose-dependent increase in GABA release in the CeA dialysate (Roberto et al., 2004a). Moreover, in dependent rats we found an increased baseline GABA tone compared to the non-dependent rats suggesting that acute and chronic ethanol increases GABA release in CeA (Roberto et al., 2004a). The CeA contains high concentrations of anti-stress neuropeptides, such as N/OFQ, known for its role in regulating anxiety- and alcohol-related behaviors (Schank et al., 2012). Previous studies have shown that N/OFQ prevents and totally reverses both the acute alcohol- and CRF-induced increases in evoked IPSC amplitudes and mIPSC frequencies opposing ethanol and CRF effects on GABA release at presynaptic site (Roberto and Siggins, 2006; Cruz et al., 2012; Ciccocioppo et al., 2014). Notably, the N/OFQ/NOP system is upregulated in CeA of ethanol-dependent rats compared to naïve controls, pointing to significant neuroadaptative changes induced by chronic ethanol exposure (Roberto and Siggins, 2006; Cruz et al., 2012). Altogether these data strongly suggest the potential of NOP agonism as a suitable approach to treat alcohol addiction. Hence, availability of small brain penetrant NOP agonists is avidly awaited to further confirm the evidence obtained with the endogenous ligand. The first nonpeptidergic brain-penetrant NOP receptor agonists developed, Ro 61-6198 (Jenck et al., 2000) and W-212393 (Teshima et al., 2005), were tested on rat alcohol-related behaviors (Economidou et al., 2006; Kuzmin et al., 2007) and circadian body temperature rhythm, respectively. Recently, a new NOP agonist, namely MT-7716, with a pharmacological profile suitable with clinical development has been synthesized. Binding and functional studies showed a high affinity and selectivity for NOP receptors. To further clarify the pharmacology of MT-7716 here we characterized its effects on the neuronal level in the CeA, comparing it with the known effects of N/OFQ in the neuronal CeA. Our results demonstrated that MT-7716 reduces evoked and spontaneous GABAergic transmission in the CeA neurons evoked by electrical stimulation in a dose dependent manner. Interestingly, the effects of MT-7716 are reversible as the GABAergic response returned to control levels after washout for all doses of the MT-7716 used, except for the highest one. Moreover, the MT-7716-induced decrease of evoked IPSP amplitude was observed in the majority (90%) of the neurons studied. Generally, MT-7716 significantly increased PPF ratios suggesting a presynaptic effect of the N/OFQ agonist on GABA release. This presynaptic effect of MT-7716 was confirmed by the significant decrease of the frequency of mIPSCs observed during MT-7716 superfusion. Importantly, the data obtained with the novel nonpeptidergic NOP agonist, are similar to our previous results using N/OFQ that dose-dependently decreased CeA GABAergic transmission, acting mostly presynaptically (Roberto and Siggins, 2006; Cruz et al., 2012).

Interestingly MT-7716, like N/OFQ reduced the mean frequency of mIPSCs, but showed a decrease of the amplitude as well, suggesting postsynaptic effects of MT-7716. Of note is that the synthetic NOP agonist MT-7716 like N/OFQ did not alter the resting membrane properties in any of the doses used, which suggests a lack of an effect on the mechanisms responsible for maintaining the RMP. In addition, MT-7716 did not alter the number of action potentials upon depolarization at any of the four concentrations tested. Importantly, [Nphe1]Nociceptin(1–13)NH2, a putative selective NOP antagonist totally prevented the MT-7716-induced inhibition of IPSP amplitudes indicating that MT-7716 exerts its effect through NOPs. Similarly, in our previous studies with N/OFQ, this same NOP antagonist blocked the N/OFQ-induced inhibition of GABAergic (Roberto and Siggins, 2006) and glutamatergic (Kallupi et al., 2013) responses. Application of the NOP antagonist did not affect the basal CeA GABAergic transmission and the ethanol-induced increase in GABAergic responses.

Finally, several lines of research have evaluated the effect of N/OFQ on ethanol-related phenomena. The activation of the NOP receptors blunts the reinforcing effects of alcohol like alcohol intake (Ciccocioppo et al., 1999), relapse to alcohol seeking (Martin-Fardon et al., 2000; Ciccocioppo et al., 2004) and conditioned place preference (Kuzmin et al., 2003). Moreover, at cellular levels, here we recapitulated that ethanol increases evoked GABA IPSPs via increased GABA release in CeA (Roberto et al., 2003), and demonstrated that the novel, synthetic nonpeptidergic NOP agonist, MT-7716 is effective in decreasing GABAergic transmission and blocking the enhancement of GABA responses induced by a maximal dose of ethanol 44 mM. In addition, MT-7716 efficiently prevented the ethanol induced increase in GABA release when applied first, and reversed the effect of ethanol when co-applied with ethanol. Thus, our data show that MT-7716, like N/OFQ, efficiently acts on the GABAergic release in CeA and opposes ethanol effects at these synapses providing rationale for developing novel therapeutics for alcoholism. Collectively, the results of our investigation will lead to a better understanding of the potential utility of employing small molecule modulators of NOP to help treat alcoholism and create the opportunity to explore the impact of manipulations of the N/OFQ system on physiological function and integrated disease-related functional correlates. Although a few NOP agonists as small molecules have been put into clinical play (Witkin et al., 2014), no clinical findings are currently available to confirm or refute hypotheses based upon preclinical evidence. Moreover, the development of other small molecules in order to penetrate the blood brain barrier, and able to elicit long lasting effects, is necessary to further investigate the alcohol effects in animal models and to perform controlled clinical trials.

## Authors contribution

Marsida Kallupi, Marisa Roberto, Roberto Ciccocioppo and Koji Teshima were responsible for the study concept and design. Marsida Kallupi, Christopher S. Oleata, George Luu and Marisa Roberto contributed to the acquisition of animal data. Marsida Kallupi, Christopher S. Oleata and Marisa Roberto assisted with the data analysis, interpretation of findings and drafted the manuscript. All authors critically reviewed the content and approved the final version for publication.

## Conflict of interest statement

The authors declare that the research was conducted in the absence of any commercial or financial relationships that could be construed as a potential conflict of interest.

## References

[B1] Allison S. Sheehy T. W. (1992). Alcohol: yesterday and today have we changed? Ala. Med. 61, 13–14, 16, 18. 1456187

[B2] Andreasen M. Hablitz J. J. (1994). Paired-pulse facilitation in the dentate gyrus: a patch-clamp study in rat hippocampus in vitro. J. Neurophysiol. 72, 326–336 7965017 10.1152/jn.1994.72.1.326

[B3] Aujla H. Cannarsa R. Romualdi P. Ciccocioppo R. Martin-Fardon R. Weiss F. (2013). Modification of anxiety-like behaviors by nociceptin/orphanin FQ (N/OFQ) and time-dependent changes in N/OFQ-NOP gene expression following ethanol withdrawal. Addict. Biol. 18, 467–479 10.1111/j.1369-1600.2012.00466.x 22804785 PMC3477306

[B4] Bonci A. Williams J. T. (1997). Increased probability of GABA release during withdrawal from morphine. J. Neurosci. 17, 796–803 8987801 10.1523/JNEUROSCI.17-02-00796.1997 PMC6573250

[B5] Braconi S. Sidhpura N. Aujla H. Martin-Fardon R. Weiss F. Ciccocioppo R. (2010). Revisiting intragastric ethanol intubation as a dependence induction method for studies of ethanol reward and motivation in rats. Alcohol. Clin. Exp. Res. 34, 538–544 10.1111/j.1530-0277.2009.01119.x 20028350 PMC2858236

[B6] Calo G. Guerrini R. Rizzi A. Salvadori S. Regoli D. (2000). Pharmacology of nociceptin and its receptor: a novel therapeutic target. Br. J. Pharmacol. 129, 1261–1283 10.1038/sj.bjp.0703219 10742280 PMC1571975

[B7] Ciccocioppo R. Angeletti S. Panocka I. Massi M. (2000). Nociceptin/orphanin FQ and drugs of abuse. Peptides 21, 1071–1080 10.1016/s0196-9781(00)00245-x 10998542

[B8] Ciccocioppo R. Biondini M. Antonelli L. Wichmann J. Jenck F. Massi M. (2002a). Reversal of stress- and CRF-induced anorexia in rats by the synthetic nociceptin/orphanin FQ receptor agonist, Ro 64-6198. Psychopharmacology 161, 113–119 10.1007/s00213-002-1020-7 11981590

[B52] Ciccocioppo R. De Guglielmo G. Hansson A. C. Ubaldi M. Kallupi M. Cruz M. T. (2014). Restraint stress alters Nociceptin/Orphanin FQ and CRF systems in the rat central Amygdala: significance for anxiety-like behaviors. J. Neurosci. 34, 363–372 10.1523/JNEUROSCI.2400-13.2014 24403138 PMC3870926

[B9] Ciccocioppo R. Economidou D. Fedeli A. Angeletti S. Weiss F. Heilig M. (2004). Attenuation of ethanol self-administration and of conditioned reinstatement of alcohol-seeking behaviour by the antiopioid peptide nociceptin/orphanin FQ in alcohol-preferring rats. Psychopharmacology 172, 170–178 10.1007/s00213-003-1645-1 14624331 PMC3035816

[B10] Ciccocioppo R. Economidou D. Rimondini R. Sommer W. Massi M. Heilig M. (2007). Buprenorphine reduces alcohol drinking through activation of the nociceptin/orphanin FQ-NOP receptor system. Biol. Psychiatry 61, 4–12 10.1016/j.biopsych.2006.01.006 16533497 PMC3035814

[B11] Ciccocioppo R. Panocka I. Polidori C. Regoli D. Massi M. (1999). Effect of nociceptin on alcohol intake in alcohol-preferring rats. Psychopharmacology (Berl) 141, 220–224 10.1007/s002130050828 9952048

[B12] Ciccocioppo R. Polidori C. Antonelli L. Salvadori S. Guerrini R. Massi M. (2002b). Pharmacological characterization of the nociceptin receptor which mediates reduction of alcohol drinking in rats. Peptides 23, 117–125 10.1016/s0196-9781(01)00587-3 11814626

[B13] Cruz M. T. Herman M. A. Kallupi M. Roberto M. (2012). Nociceptin/orphanin FQ blockade of corticotropin-releasing factor-induced gamma-aminobutyric acid release in central amygdala is enhanced after chronic ethanol exposure. Biol. Psychiatry 71, 666–676 10.1016/j.biopsych.2011.10.032 22153590 PMC3838304

[B14] Dahchour A. Lallemand F. Ward R. J. De Witte P. (2005). Production of reactive oxygen species following acute ethanol or acetaldehyde and its reduction by acamprosate in chronically alcoholized rats. Eur. J. Pharmacol. 520, 51–58 10.1016/j.ejphar.2005.07.012 16135364

[B15] Economidou D. Fedeli A. Fardon R. M. Weiss F. Massi M. Ciccocioppo R. (2006). Effect of novel nociceptin/orphanin FQ-NOP receptor ligands on ethanol drinking in alcohol-preferring msP rats. Peptides 27, 3299–3306 10.1016/j.peptides.2006.09.007 17097763 PMC1847604

[B16] Economidou D. Hansson A. C. Weiss F. Terasmaa A. Sommer W. H. Cippitelli A. (2008). Dysregulation of nociceptin/orphanin FQ activity in the amygdala is linked to excessive alcohol drinking in the rat. Biol. Psychiatry 64, 211–218 10.1016/j.biopsych.2008.02.004 18367152 PMC4275225

[B17] Gavioli E. C. Calo G. (2013). Nociceptin/orphanin FQ receptor antagonists as innovative antidepressant drugs. Pharmacol. Ther. 140, 10–25 10.1016/j.pharmthera.2013.05.008 23711793

[B18] Gilpin N. W. Misra K. Herman M. A. Cruz M. T. Koob G. F. Roberto M. (2011). Neuropeptide Y opposes alcohol effects on gamma-aminobutyric acid release in amygdala and blocks the transition to alcohol dependence. Biol. Psychiatry 69, 1091–1099 10.1016/j.biopsych.2011.02.004 21459365 PMC3090491

[B19] Greenfield T. K. Ye Y. Kerr W. Bond J. Rehm J. Giesbrecht N. (2009). Externalities from alcohol consumption in the 2005 US National Alcohol Survey: implications for policy. Int. J. Environ. Res. Public Health 6, 3205–3224 10.3390/ijerph6123205 20049257 PMC2800345

[B20] Haubensak W. Kunwar P. S. Cai H. Ciocchi S. Wall N. R. Ponnusamy R. (2010). Genetic dissection of an amygdala microcircuit that gates conditioned fear. Nature 468, 270–276 10.1038/nature09553 21068836 PMC3597095

[B21] Herman M. A. Kallupi M. Luu G. Oleata C. S. Heilig M. Koob G. F. (2013). Enhanced GABAergic transmission in the central nucleus of the amygdala of genetically selected Marchigian Sardinian rats: alcohol and CRF effects. Neuropharmacology 67C, 337–348 10.1016/j.neuropharm.2012.11.026 23220399 PMC3562384

[B22] Higley A. E. Koob G. F. Mason B. J. (2012). Treatment of alcohol dependence with drug antagonists of the stress response. Alcohol Res. 34, 516–521 23584117 10.35946/arcr.v34.4.15 PMC3860394

[B23] Jenck F. Moreau J. L. Martin J. R. Kilpatrick G. J. Reinscheid R. K. Monsma F. J. (1997). Orphanin FQ acts as an anxiolytic to attenuate behavioral responses to stress. Proc. Natl. Acad. Sci. U S A 94, 14854–14858 10.1073/pnas.94.26.14854 9405703 PMC25127

[B24] Jenck F. Wichmann J. Dautzenberg F. M. Moreau J. L. Ouagazzal A. M. Martin J. R. (2000). A synthetic agonist at the orphanin FQ/nociceptin receptor ORL1: anxiolytic profile in the rat. Proc. Natl. Acad. Sci. U S A 97, 4938–4943 10.1073/pnas.090514397 10758169 PMC18336

[B25] Kallupi M. Varodayan F. P. Oleata C. S. Correia D. Luu G. Roberto M. (2013). Nociceptin/orphanin FQ decreases glutamate transmission and blocks ethanol-induced effects in the central amygdala of naive and ethanol-dependent rats. Neuropsychopharmacology [Epub ahead of print]. 10.1038/npp.2013.308 24169802 PMC3957102

[B26] Koob G. F. (2009). Brain stress systems in the amygdala and addiction. Brain Res. 1293, 61–75 10.1016/j.brainres.2009.03.038 19332030 PMC2774745

[B27] Koob G. F. Le Moal M. (1997). Drug abuse: hedonic homeostatic dysregulation. Science 278, 52–58 10.1126/science.278.5335.52 9311926

[B28] Koob G. F. Mason B. J. De Witte P. Littleton J. Siggins G. R. (2002). Potential neuroprotective effects of acamprosate. Alcohol. Clin. Exp. Res. 26, 586–592 10.1111/j.1530-0277.2002.tb02578.x 11981137

[B29] Koster A. Montkowski A. Schulz S. Stube E. M. Knaudt K. Jenck F. (1999). Targeted disruption of the orphanin FQ/nociceptin gene increases stress susceptibility and impairs stress adaptation in mice. Proc. Natl. Acad. Sci. U S A 96, 10444–10449 10.1073/pnas.96.18.10444 10468628 PMC17908

[B30] Kuzmin A. Kreek M. J. Bakalkin G. Liljequist S. (2007). The nociceptin/orphanin FQ receptor agonist Ro 64-6198 reduces alcohol self-administration and prevents relapse-like alcohol drinking. Neuropsychopharmacology 32, 902–910 10.1038/sj.npp.1301169 16880770

[B31] Kuzmin A. Sandin J. Terenius L. Ogren S. O. (2003). Acquisition, expression and reinstatement of ethanol-induced conditioned place preference in mice: effects of opioid receptor-like 1 receptor agonists and naloxone. J. Pharmacol. Exp. Ther. 304, 310–318 10.1124/jpet.102.041350 12490606

[B32] Lambert D. G. (2008). The nociceptin/orphanin FQ receptor: a target with broad therapeutic potential. Nat. Rev. Drug Discov. 7, 694–710 10.1038/nrd2572 18670432

[B33] Mann K. Kiefer F. Spanagel R. Littleton J. (2008). Acamprosate: recent findings and future research directions. Alcohol. Clin. Exp. Res. 32, 1105–1110 10.1111/j.1530-0277.2008.00690.x 18540918

[B34] Mann K. Lehert P. Morgan M. Y. (2004). The efficacy of acamprosate in the maintenance of abstinence in alcohol-dependent individuals: results of a meta-analysis. Alcohol. Clin. Exp. Res. 28, 51–63 10.1097/01.alc.0000108656.81563.05 14745302

[B35] Martin-Fardon R. Ciccocioppo R. Massi M. Weiss F. (2000). Nociceptin prevents stress-induced ethanol- but not cocaine-seeking behavior in rats. Neuroreport 11, 1939–1943 10.1097/00001756-200006260-00026 10884047

[B36] Martin-Fardon R. Zorrilla E. P. Ciccocioppo R. Weiss F. (2010). Role of innate and drug-induced dysregulation of brain stress and arousal systems in addiction: focus on corticotropin-releasing factor, nociceptin/orphanin FQ and orexin/hypocretin. Brain Res. 1314, 145–161 10.1016/j.brainres.2009.12.027 20026088 PMC2819635

[B37] Meunier J. C. (1997). Nociceptin/orphanin FQ and the opioid receptor-like ORL1 receptor. Eur. J. Pharmacol. 340, 1–15 10.1016/s0014-2999(97)01411-8 9527501

[B38] Meunier J. C. Mollereau C. Toll L. Suaudeau C. Moisand C. Alvinerie P. (1995). Isolation and structure of the endogenous agonist of opioid receptor-like ORL1 receptor. Nature 377, 532–535 10.1038/377532a0 7566152

[B39] Mohapatra S. Patra J. Popova S. Duhig A. Rehm J. (2010). Social cost of heavy drinking and alcohol dependence in high-income countries. Int. J. Public Health 55, 149–157 10.1007/s00038-009-0108-9 20024666

[B40] Reinscheid R. K. Nothacker H. P. Bourson A. Ardati A. Henningsen R. A. Bunzow J. R. (1995). Orphanin FQ: a neuropeptide that activates an opioidlike G protein-coupled receptor. Science 270, 792–794 10.1126/science.270.5237.792 7481766

[B41] Reinscheid R. K. Nothacker H. P. Civelli O. (1999). Orphan receptors and the concept of reverse physiology: discovery of the novel neuropeptide orphanin FQ/nociceptin. Results Probl. Cell Differ. 26, 193–214 10.1007/978-3-540-49421-8_9 10453465

[B42] Roberto M. Madamba S. G. Moore S. D. Tallent M. K. Siggins G. R. (2003). Ethanol increases GABAergic transmission at both pre- and postsynaptic sites in rat central amygdala neurons. Proc. Natl. Acad. Sci. U S A 100, 2053–2058 10.1073/pnas.0437926100 12566570 PMC149957

[B43] Roberto M. Madamba S. G. Stouffer D. G. Parsons L. H. Siggins G. R. (2004a). Increased GABA release in the central amygdala of ethanol-dependent rats. J. Neurosci. 24, 10159–10166 10.1523/jneurosci.3004-04.2004 15537886 PMC6730176

[B44] Roberto M. Schweitzer P. Madamba S. G. Stouffer D. G. Parsons L. H. Siggins G. R. (2004b). Acute and chronic ethanol alter glutamatergic transmission in rat central amygdala: an in vitro and in vivo analysis. J. Neurosci. 24, 1594–1603 10.1523/jneurosci.5077-03.2004 14973247 PMC6730456

[B45] Roberto M. Siggins G. R. (2006). Nociceptin/orphanin FQ presynaptically decreases GABAergic transmission and blocks the ethanol-induced increase of GABA release in central amygdala. Proc. Natl. Acad. Sci. U S A 103, 9715–9720 10.1073/pnas.0601899103 16788074 PMC1480472

[B46] Schank J. R. Ryabinin A. E. Giardino W. J. Ciccocioppo R. Heilig M. (2012). Stress-related neuropeptides and addictive behaviors: beyond the usual suspects. Neuron 76, 192–208 10.1016/j.neuron.2012.09.026 23040815 PMC3495179

[B47] Spanagel R. Vengeliene V. (2013). New pharmacological treatment strategies for relapse prevention. Curr. Top. Behav. Neurosci. 13, 583–609 10.1007/7854_2012_205 22389180

[B48] Teshima K. Minoguchi M. Tounai S. Ashimori A. Eguchi J. Allen C. N. (2005). Nonphotic entrainment of the circadian body temperature rhythm by the selective ORL1 receptor agonist W-212393 in rats. Br. J. Pharmacol. 146, 33–40 10.1038/sj.bjp.0706311 15980870 PMC1576254

[B49] Ubaldi M. Bifone A. Ciccocioppo R. (2013). Translational approach to develop novel medications on alcohol addiction: focus on neuropeptides. Curr. Opin. Neurobiol. 23, 684–691 10.1016/j.conb.2013.04.009 23648086 PMC3735833

[B50] Umhau J. C. Schwandt M. L. Usala J. Geyer C. Singley E. George D. T. (2011). Pharmacologically induced alcohol craving in treatment seeking alcoholics correlates with alcoholism severity, but is insensitive to acamprosate. Neuropsychopharmacology 36, 1178–1186 10.1038/npp.2010.253 21289601 PMC3077446

[B51] Witkin J. M. Statnick M. A. Rorick-Kehn L. M. Pintar J. E. Ansonoff M. Chen Y. (2014). The biology of Nociceptin/Orphanin FQ (N/OFQ) related to obesity, stress, anxiety, mood and drug dependence. Pharmacol. Ther. 141, 283–299 10.1016/j.pharmthera.2013.10.011 24189487 PMC5098338

